# Constructing the concept of healthy ageing and examining its association with loneliness in older adults

**DOI:** 10.1186/s12877-023-04019-5

**Published:** 2023-05-25

**Authors:** Ivy Yan Zhao, Mu-Hsing Ho, Stefanos Tyrovolas, Sasha Yuanjie Deng, Jed Montayre, Alex Molassiotis

**Affiliations:** 1 WHOCC Centre for Community Health Services, School of Nursing, the Hong Kong Polytechnic University, Hong Kong, SAR P. R. China; 2 School of Nursing, the Hong Kong Polytechnic University, Hong Kong, SAR P. R. China; 3 School of Nursing, LKS Faculty of Medicine, The University of Hong Kong, Pokfulam, Hong Kong SAR P. R. China; 4 Office of the Vice-Chancellor, College of Arts, Humanities and Education, University of Derby, Derby, UK

**Keywords:** Ageing, Healthy ageing, Health services, Activities of daily living, Loneliness, Gerontology

## Abstract

**Background:**

World Health Organization (WHO) has defined healthy ageing by highlighting five functional ability domains to (meet basic needs, make decisions, be mobile, build and maintain relationships, and contribute to society), which also emphasized the importance of addressing loneliness as priorities within United Nations Decade of Healthy Ageing initiative. However, the level and determinants of healthy ageing and its association with loneliness are rarely examined. This study aimed to construct a healthy ageing index to verify the WHO healthy ageing framework, measure five domains of functional ability of older adults and examine the relationship between functional ability domains and loneliness.

**Methods:**

A total of 10,746 older adults from the 2018 China Health and Retirement Longitudinal Study (CHARLS) were included. A healthy ageing index ranging from 0 to 17 was constructed using 17 components related to functional ability domains. Univariate and multivariate logistic regression analyses were utilized to determine the association between loneliness and healthy ageing. The STROBE guidelines with the RECORD statement for observational studies using routinely collected health data were observed.

**Results:**

The factor analysis verified the five functional ability domains for healthy ageing. After adjusting for confounders, being mobile, building and maintaining relationships, and learning, growing and making decisions were significantly associated with lesser loneliness among participants.

**Conclusions:**

The healthy ageing index of this study can be utilized and further modified with respect to large-scale research with relevant healthy ageing topics. Our findings will support healthcare professionals to provide patient-centered care when identifying their comprehensive abilities and needs.

## Background

China is one of the fastest ageing countries, where the number of individuals aged 60 years and above is expected to reach 397 million in 2040 [1]. The emerging demographic shift compounded by chronic conditions in later life will further stress the already overloaded healthcare and social services [2]. In terms of introducing a household registration system (hukou) by the Chinese government in the 1950s [3] and high urbanization [4], the rural-urban disparities in healthy ageing were prevalent in China [5]. Many older adults living in rural areas were left behind due to the urbanization and domestic emigration of their adult children [6], which were linked to older adults’ experiences of loneliness [5]. Loneliness is defined as subjective perceptions of distress and being socially isolated due to unsatisfied social relationships one has received [7]. Loneliness impacts multiple aspects of people’s lives.

According to the World Health Organization (WHO), loneliness is prominent in old age and has been an increasing challenge for healthy ageing [8]. Loneliness might have negative effects on the physical and psychological aspects of health, quality of life and longevity of older adults [9, 10]. Conversely, older adults with a variety of physical and psychosocial health problems, such as cognitive decline, chronic disease, physical disability, depression, poor family relationships, and inadequate social support are more prone to experiencing loneliness [11, 12].

In 2015, the WHO released the World Reported on Ageing and Health and defined Healthy Ageing as “*the process of developing and maintaining the functional ability that enables well-being in older age*” [13]. The report highlighted that functional abilities are important considerations for healthy ageing, instead of focusing on the absence of disease [13]. Functional abilities were further defined comprehensively by the WHO as older adults’ abilities in five domains, namely: to meet their basic needs, make decisions, be mobile, build and maintain relationships and contribute to society [13]. In response to the increasing challenge of population ageing, the Chinese central government has formally released the 14th Five-Year Plan for Healthy Age in 2022, which emphasizes that research on healthy ageing is a key component of national strategies to meet the goals of Healthy China 2030 [14].

Previous research into healthy ageing mainly focused on the functional abilities to meet basic needs and to be mobile [15], while the other three domains of functional abilities (to make decisions, to build and maintain relationships and to contribute to society) were less addressed. For example, a systematic review and meta-analysis of longitudinal cohort studies revealed that physical activity is positively associated with healthy ageing [16]. There were limited intervention studies designed to investigate other domains of functional abilities, for example with psycho-social foci [15]. Only a qualitative study in China has been identified to adopt the WHO healthy ageing framework into their community ageing care centers’ services and suggested that caregivers followed the framework and developed more strategic daily activities for older residents to promote their well-being [17], however, the WHO healthy ageing framework (five domains functional ability) was less verified so far. Although the WHO has defined healthy ageing to encompass psycho-social wellbeing, and both international and national agencies have highlighted the importance of addressing loneliness for holistic healthy ageing, to date, the association between loneliness and healthy ageing has been underestimated especially the relationships between loneliness and each functional ability domain [5]. Moreover, there is a limited index focusing on a nationally representative population to systematically evaluate healthy ageing [15]. In this study, we constructed a healthy ageing index to verify the WHO healthy ageing framework, measure five domains of functional ability of older adults and examine the relationship between functional ability domains and loneliness.

## Materials and methods

### Database and sample

Data used in this study were from the 2018 China Health and Retirement Longitudinal Study (CHARLS), a nationally representative panel survey among community residents aged 45 years and older that has been collecting information on the sociodemographic, economic and health status of adults since 2011. CHARLS utilized multi-stage stratified Probability Proportionate to Size sampling (PPS) to generate a sampling framework. A section of filter at the beginning of the CHARLS questionnaire was adopted to eliminate invalid questionnaires to guarantee the quality and representativeness of the data. The latest data of CHARLS (wave 4, 2018) involved 19,816 individuals who were from 150 districts of 28 provinces in China [18]. All data collected are maintained by the Institute of Social Science Survey of Peking University and have been publicly released on the website of CHARLS project (http://charls.pku.edu.cn). Registration for accessing the database is approved. According to the purpose of this study, participants aged 60 and above were included. With our study focusing on constructing a healthy ageing index and examining its relationship with loneliness particularly among those who were aged over 60 years, the CHARLS sampling method allowed us to test the national-level representative of older people. Thus, a weighted analysis was not applied because of our focus on a specific age group in the CHARLS with small subsample sizes (54.2% of all respondents in the database). The STROBE guidelines with the RECORD statement for observational studies using routinely collected health data were observed.

### Ethical approval

The ethics approval of the Peking University ethics committee was obtained. The original study obtained informed consent from all respondents. The current study has obtained the exemption from review in December 2020.

### Variable description

Demographic information including age, gender, residential status, living in rural areas or not, and marital status was included in the analysis. Age was coded as a continuous variable and the remaining demographic data were analyzed as categorical variables. Those participants who lived in the center of the city/town and the combination zone between urban and rural areas were coded as living in non-rural areas in this study.

### Selected variables for the evaluation of healthy ageing

As mentioned above, the WHO Healthy Ageing framework outlined five domains of functional ability in older age [19], and based on that, the study team selected and agreed on a list of 17 components covering older adults’ abilities to meet basic needs, be mobile, make decisions, build and maintain relationships and contribute to society [20]. All components were also assessed by the study team across published studies having consistency in at least three studies. The creation of a healthy ageing index was adopted to accurately undertake a multi-variable statistical analysis and to avoid co-linearity between the previously mentioned components [21]. Our methodological approach was based on previously published works in the area of healthy ageing and frailty and the construction of related indices [22–24]. Specifically, individual ratings (from 0 to 1) in each of the 17 components were assigned according to their positive or negative impact on healthy ageing. Specifically, for the 4-scale components (i.e., cannot do it/ have difficulty and need help/ have difficulty but can still do it/ have no difficulty), the score 0 was utilized if the individual ability was in the lowest level, scores 0.33, 0.66 and 1 were assigned for each higher response level (Table 1). The generated healthy ageing index was demonstrated as the cumulative score of the 17 components (theoretical range 0–17). Higher scores indicated greater performances in healthy ageing.

**Table 1:** Description and coding of the healthy ageing (functional ability) index (total theoretical score 0–17)

Characteristics	Cut points and scores
Dressing	Cannot do it = 0, have difficulty and need help = 0.33, have difficulty but can still do it = 0.66, have no difficulty = 1
Bathing or showering	Cannot do it = 0, have difficulty and need help = 0.33, have difficulty but can still do it = 0.66, have no difficulty = 1
Using the toilet, including getting up and down	Cannot do it = 0, have difficulty and need help = 0.33, have difficulty but can still do it = 0.66, have no difficulty = 1
Difficulties with shopping for groceries due to the health and memory problems	Cannot do it = 0, have difficulty and need help = 0.33, have difficulty but can still do it = 0.66, have no difficulty = 1
Eating food by oneself when it is ready	Cannot do it = 0, have difficulty and need help = 0.33, have difficulty but can still do it = 0.66, have no difficulty = 1
Getting into or out of bed	Cannot do it = 0, have difficulty and need help = 0.33, have difficulty but can still do it = 0.66, have no difficulty = 1
Getting up from a chair after sitting for a long period	Cannot do it = 0, have difficulty and need help = 0.33, have difficulty but can still do it = 0.66, have no difficulty = 1
Managing money	Cannot do it = 0, have difficulty and need help = 0.33, have difficulty but can still do it = 0.66, have no difficulty = 1
Taking the right portion of medication right on time	Cannot do it = 0, have difficulty and need help = 0.33, have difficulty but can still do it = 0.66, have no difficulty = 1
Troubled with body pain	Cannot do it = 0, have difficulty and need help = 0.33, have difficulty but can still do it = 0.66, have no difficulty = 1
Running or jogging about1km	Cannot do it = 0, have difficulty and need help = 0.33, have difficulty but can still do it = 0.66, have no difficulty = 1
Self-reported health status	Very poor = 0, poor = 0.25, fair = 0.5, good = 0.75, very good = 1
Interacted with friends	No = 0, yes = 1
Joined a community-related organization or group	No = 0, yes = 1
Used the internet in the last month	No = 0, yes = 1
Did nonfarm work for at least one hour in last month	No = 0, yes = 1
Employed as a farmer	No = 0, yes = 1

### Loneliness

Loneliness was measured by a single question ‘Have you felt lonely during the last week?’ in the Center for Epidemiologic Studies Depression Scale (CES-D) which has been generally utilized to measure depressive symptoms. The single question of measuring loneliness has been proven valid and appropriate for use among the ageing population [25]. There are four answer options: “rarely or none of the time (< 1 day)”, “some or a little of the time (1–2 days)”, “occasionally or a moderate amount of time (3–4 days)”, or “most or all of the time (5–7 days)”. In this study, we recoded the “rarely or none of the time” into 0 (not lonely) and other options as 1 (lonely). A binary variable was created to contrast older adults with and without loneliness [26].

### Statistical analysis

Descriptive analysis, including mean, standard deviation (SD), frequency and percentage was utilized to describe the distribution of demographic information and the functional ability in five domains. Factor analysis using the principal components method was adopted to define the degree to which the composed index provided a correct reflection of the multi-dimensionality of healthy ageing [24]. Principal components with eigenvalues higher than 1.0 were maintained, which is a threshold generally used as a cut-off to identify meaningful patterns. The components generated in the study results were interpreted based on the variables with loadings above 0.3. Univariate and multivariate logistic regression analyses were utilized to examine the association between loneliness and each component of the healthy ageing index. All data analyses were performed by the IBM SPSS Statistics for Windows Version 26.0 (IBM Inc., Armonk, NY, USA), and a *p* < 0.05 was considered significant in this study.

## Results

Of the 19,816 people eligible to take part in the 2018 CHARLS study, 10,746 participants (54.2%) were finally analyzed after removing the samples with missing key variables. The mean age of the participants was 69.10 ± 7.15, and 51.3% of the participants were females. The prevalence of loneliness among older adults was 28.3%. A higher proportion of participants lived in their own homes (n = 10,528, 98%) and were married (n = 8,342, 77.6%). Most of them came from rural areas (n = 7,864, 73.2%). Table 2 presents the detailed demographic information of participants. The mean healthy ageing score was 9.50 ± 2.28 (Median 9.92); for females, the mean score was 9.54 ± 2.22 (Median 9.92) and for males 9.46 ± 2.34 (Median 9.75) (*p = 0.051*).

**Table 2:** Demographic information of participants (N = 10,746)

	n (%) / Mean ± SD
Age	69.10 ± 7.15
Gender	
- Male	5,232 (48.7)
- Female	5,514 (51.3)
Residential status	
- Family house	10,528 (98)
- Nursing home	47 (0.4)
- Hospital	12 (0.1)
- Other	159 (1.5)
Living in rural areas or not	
- Rural areas	7,864 (73.2)
- Non-rural areas	2,882 (26.8)
Marital status	
- Married	8,342 (77.6)
- Divorced/separated	134 (1.2)
- Widower/widowed	2,196 (20.4)
- Never married	74 (0.7)

In terms of the five domains of functional ability of the participants, the reported abilities to meet their basic needs and to learn, grow and make decisions had better scores than the ability to be mobile (Table 3). Among them, interacting with friends was the most common way (n = 4,510, 42.2%) of building and maintaining relationships with others, while joining a community-related organization or group and using the internet in the last month were only 7.5% and 0.6% respectively. There were small proportions of participants employed to do non-farm (n = 1,388, 13%) and farm work (n = 333, 3.1%) (Table 3**)**.

**Table 3:** Five domains functional ability of participants

	No. of response	n (%) / Mean ± SD
**Meet their basic needs**		
Dressing^1^	8,480	3.81 ± 0.56
Bathing or showering^1^	8,480	3.71 ± 0.73
Using the toilet, including getting up and down^1^	8,480	3.71 ± 0.65
Eating food by oneself when it is ready^1^	8,480	3.92 ± 0.40
Getting into or out of bed^1^	8,480	3.82 ± 0.52
Getting up from a chair after sitting for a long period^1^	10,693	3.55 ± 0.67
Difficulties with shopping for groceries due to the health and memory problems ^1^	10,692	3.66 ± 0.89
**Learn, grow and make decision**		
Managing money^1^	10,692	3.63 ± 0.92
Taking the right portion of medication right on time ^1^	10,692	3.84 ± 0.57
**Be mobile**		
Troubled with body pain^2^	10,699	3.74 ± 1.33
Running or jogging about1km^1^	10,693	2.25 ± 1.41
Self-reported health status^3^	9,781	2.92 ± 1.01
**Built and maintain relationships**		
Interacted with friends	10,697	
- Yes		4510 (42.2)
Joined a community-related organization or group	10,697	
- Yes		803 (7.5)
Used the internet in the last month	10,697	
- Yes		642 (0.6)
**Contribute to society**		
Did nonfarm work for at least one hour in last month	10,690	
- Yes		1,388 (13)
Employed as a farmer	10,358	
- Yes		333 (3.1)

Note:

^1^. Score range: 1–4 from very difficult to no difficult;

^2^. Score range: 1–5 from very pain to no pain;

^3^. Score range: 1–5 from very poor to very good

Table 4 presents the component loading and the eigenvalues from the factor analysis. The size of eigenvalues strongly suggests that healthy ageing has a five-dimensional structure, explaining 52% of the total variance of the information. Cumulative variance explained (%) for each domain was 25%, 33%, 40%, 46% and 52%, respectively. In particular, the first component’s dominant variables were the ability to meet their activities of daily living. The second component’s dominant variables were the self-reported status of health, doing physical activities and problems with body pain. The third component’s dominant variables were interaction with friends, joining groups and connecting with others via the internet. The fourth component’s dominant variables were employed to do non-farm works or farm works. Finally, the dominant variables included in the fifth component were the ability to decidein managing money or taking medications.

**Table 4:** Factor loadings of the variables included in the functional ability index. In each factor only the components with values > 0.3 are included in the table

	Meet their basic needs	Be mobile	Build and maintain relationships	Contribute to society	Learn, grow and make decision
Getting into or out of bed	0.742	-	-	-	-
Bathing or showering	0.735	-	-	-	-
Dressing	0.727	-	-	-	-
Using the toilet, including getting up and down	0.659	-	-	-	-
Difficulties with shopping for groceries due to the health and memory problems	0.647				
Getting up from a chair after sitting for a long period	0.574	-	-	-	-
Eating food by oneself when it is ready	0.562	-	-	-	-
Troubled with body pain	-	0.379	-	-	-
Running or jogging about1km	-	0.466	-	-	-
Self-reported health status	-	0.449	-	-	-
Used the internet in the last month	-	-	0.421	-	-
Joined a community-related organization or group	-	-	0.517	-	-
Interacted with friends	-	-	0.466	-	-
Did nonfarm work for at least one hour in last month	-	-	-	0.369	-
Employed as a farmer	-	-	-	0.458	-
Managing money	-	-	-	-	− 0.366
Taking the right portion of medication right on time	-	-	-	-	− 0.300
*Cumulative Variance explained* (%)	25%	33%	40%	46%	52%

To examine the association between loneliness status and healthy ageing, the five components of the healthy ageing index were tested by univariate and multivariate logistic regression analyses. After adjusting the factors of age, gender and marital status, results from the multivariate logistic regression model (Table 5) showed that the components of being mobile (adjusted odds ratio = 0.567, 95% confidence interval, 0.529–0.608, *p* < 0.001), building and maintaining relationships (adjusted odds ratio = 0.906, 95% confidence interval, 0.846–0.971, *p* = 0.005), and learning, growing and making decisions (adjusted odds ratio = 0.657, 95% confidence interval, 0.575–0.751, *p* < 0.001) were significantly associated with loneliness among older adults.

**Table 5:** Binary logistic regression models evaluated the association between loneliness and the functional ability index (N = 9,335)

	Felt lonely or not				
	Crude OR (95% CI)	*p*	Adjusted OR (95% CI)	*p*	
Gender	1.461(1.339–1.594)	***< 0.001***	1.026 (0.932–1.13)	0.599	
Age	1.013(1.007–1.02)	***< 0.001***	0.982 (0.974–0.989)	***< 0.001***	
Marital status (ref: married)		***< 0.001***	-	***< 0.001***	
-Married	1.00	-	-	-	
-Separated/divorced	2.597(1.812–3.722)	***< 0.001***	0.41(0.241–0.696)	***0.001***	
-Widowed	2.513(2.257–2.798)	***< 0.001***	1.085(0.57–2.065)	0.805	
-Never married	2.469(1.478–4.124)	***0.001***	1.01(0.588–1.733)	0.972	
**Functional ability**					
Meet their basic needs	1.08(1.055–1.105)	***< 0.001***	0.993(0.966–1.021)	0.623	
Be mobile	0.535(0.503–0.568)	***< 0.001***	0.567(0.529–0.608)	***< 0.001***	
Build and maintain relationships	0.814(0.762–0.871)	***< 0.001***	0.906(0.846–0.971)	**0.005**	
Contribute to society	0.742(0.656–0.84)	***< 0.001***	0.921(0.811–1.047)	0.209	
Learn, grow and make decision	0.475(0.419–0.539)	***< 0.001***	0.657(0.575–0.751)	***< 0.001***	

Adjusted for age, gender, marital status. Statistically significant results as *p* < 0.05 are in bold

## Discussion

This is one of the few studies that evaluated the level of healthy ageing and its determinants by constructing a healthy ageing index to measure the functional abilities of older adults. It adds knowledge to the previous study conducted by Sanchez-Niubo and his colleagues [15], who developed a scale for measuring healthy ageing via a logistic regression framework with good validity and reliability. The multidimensionality of healthy ageing was evident throughout the domains of meeting basic needs, making decisions, being mobile, building and maintaining relationships and contributing to society, which supported the WHO definition of healthy ageing [13]. We observed that three functional ability components, namely: ‘be mobile’, ‘build and maintain relationships’, and ‘learn, grow and make decisions’, were significantly associated with lesser loneliness in older adults, which are consistent with the existing studies that physical activity [27], social connection [28] and acquiring technical skills and knowledge [29] are significantly associated with decreased loneliness. These findings are of great importance for healthy ageing since targeted policies and healthcare services are required to address the challenges of demographic ageing and loneliness.

Seventeen components were selected in this study to verify WHO five domains of functional ability. Seven components related to functional limitations were selected to check people’s ability to meet their basic needs, and three components were included to assess being mobile. The components of the two domains are consistent with most existing studies that commonly used standards for assessing the functional ability of older adults are Activities of Daily Living (ADL) [30] and Instrumental Activities of Daily Living (IADL) [31, 32]. Three components of the domains of being mobile are also aligned with the documented evidence on managing comorbidities, pain levels and the level of physical activity among older adults to achieve healthy ageing [33]. However, a discrepancy in the definition of functional ability has been identified between existing studies/measurement tools for the functional ability and the WHO classification. According to the International Classification of Functioning, Disability and Health (ICF) of WHO, functional ability consists of the intrinsic capacity of the individual, relevant environmental characteristics and the interaction between these two factors [34]. Based on that, this study included five domains of functional ability intending to evaluate healthy ageing comprehensively to enable the whole well-being of older adults, while the current measurement tools (e.g., ADL, IADL) are focusing on physical health only. The findings of this study are aligned with the WHO Guidelines on Integrated Care for Older People (ICOPE) proposing evidence-based recommendations for healthcare professionals to place the needs and preferences of older adults at the center of coordinated care models (World Health Organization, 2017). Future research could also consider evaluating long-term healthy ageing by constructing the concept of healthy ageing based on cohort studies.

Besides the domains of physical health, we checked the domains of being able to build and maintain relationships comprehensively through interpersonal, group, institutional and connecting using the internet levels. The selection of these components is important for improving comprehensive gerontological care and aligned with the finding of a study that older adults valued the functioning of social integration to achieve healthy ageing [35]. In particular, we included the item of internet use, since the experiences of using the internet and apps (e.g., WhatsApp or WeChat) to connect with people and share knowledge were significantly related to less loneliness in older Chinese adults [29]. Two components of working on the farm and non-farm works were included to assess the ability to contribute to society, which is aligned with Stephens, Breheny and Mansvelt [35]’s study, the functioning of remaining in the workforce and contributing their skills and knowledge to society were regarded as important to promote healthy ageing in the lives of older adults. In addition, a study in Japan reported that older adults with farm work experience required less long-term care prior to death, suggesting that agricultural and physical activities promote health [36]. Lastly, older adult’s ability to make decisions in managing money and taking medications were included since decision making (such as making choices during financial risk-taking) was reported to be related to alterations in the affective and motivational circuits of the ageing brain [37]. In the UK, the effect of a tablet computer training intervention was examined for cognitive function and reported that learning new skills in later life, including those related to adopting new technologies (tablet training) was associated with improved processing speed [38]. More research is needed on exploring the relationship between learning, growing and healthy ageing.

The social participation and integration of older adults are important aspects of healthy ageing. Feelings of loneliness were identified to be related to satisfaction with one’s social network [39] and this is in accordance with McCormack and McCance’s [40] framework of Person-Centered Practice for Nurses which steers the creation of an inclusive and robust relationship with patients to improve the quality of health care. A novel finding of this study demonstrated a significant association between the ability to ‘learn, grow and make decisions’ and loneliness while existing studies examining this relationship are sparse. Although decision-makingrelated cognitive function was associated with loneliness among older Chinese adults [41, 42], more studies are needed to investigate the effect of increasing older adults’ ability to learn, grow, and make decisions on their experiences of loneliness. Our study findings will inform the service providers and policymakers to create livable, engaged social neighborhoods, and accessible community health services for older residents to support healthy ageing. More evidence are need on the relationships between each item under the five domains and loneliness. Community health services should be tailored catering to older residents’ needs and preferences, which will be beneficial to promote their satisfaction of neighborhood environment.

### Limitations and strengths

The limitations of this study are: (1) items selected from the CHARLS database may not comprehensively represent the healthy ageing (functional ability) index defined in the current study; (2) the loneliness was measured by a single item of the CES-D which may result in higher false positive and false negative rates; (3) potential confounding variables, such as some biological indicators and disease status may not be included due to the limitation of secondary data analysis and the retrospective dataset; (4) As the WHO healthy ageing functional ability domains at this stage provide an inclusive yet loose definition, although we have carefully selected questions based on the WHO definition of each domain, the interpretations of functional ability could vary among different researchers Nevertheless, using data from a nationally representative panel survey with a large sample size provides meaningful population-based evidence in the healthy ageing investigation.

## Conclusion

A comprehensive literature review in addition to WHO healthy ageing framework was used to identify the most appropriate variables. Moreover, a rigorous statistical approach was applied in this study. The healthy ageing (functional ability) index confirmed in this study can be utilized and further modified with respect to large-scale research with relevant healthy ageing topics. The present findings estimated the level of healthy ageing and verified the appropriateness of the five domains of functional ability in the assessment of healthy ageing through a multi-dimensional approach. The ability to be mobile, to build and maintain relationships, and to learn, grow and make decisions, were significantly associated with less loneliness in older adults, which further determined the association between healthy ageing and loneliness. Further intervention research on improving healthy ageing for reducing loneliness among diverse ethnic groups is needed to improve our understanding of healthy ageing and its association with loneliness.

## Data Availability

The CHARLS is an ongoing national, longitudinal survey for residents aged 45 and above in China, sponsored by the National Development Research Institute of Peking University and jointly implemented by the China Social Science Survey Center of Peking University and the Youth League Committee of Peking University. All data collected in the CHARLS are maintained at the National School of Development of Peking University http://charls.pku.edu.cn.

## List of Abbreviations

CHARLS: China Health and Retirement Longitudinal Study
PPS: Probability Proportionate to Size
CES-D: Center for Epidemiologic Studies Depression Scale
SD: Standard deviation
ADL: Activities of Daily Living
IADL: Instrumental Activities of Daily Living
ICOPE: Integrated Care for Older People
WHO: World Health Organization
